# What are “good outcomes” in public mental health settings? A qualitative exploration of clients’ and therapists’ experiences

**DOI:** 10.1186/s13033-017-0119-5

**Published:** 2017-01-14

**Authors:** Christian Moltu, Jon Stefansen, Jan Christian Nøtnes, Åse Skjølberg, Marius Veseth

**Affiliations:** 1 Department of Psychiatry, District General Hospital of Førde, Førde, Norway; 2 Department of Clinical Psychology, University of Bergen, Bergen, Norway; 3 Division of Mental Health and Addictions, Oslo University Hospital, Oslo, Norway

**Keywords:** Routine Outcomes, Monitoring Systems, Outcome, Recovery

## Abstract

**Background:**

The mental health field sees a surge of interest in Routine Outcome Monitoring, mandated by a wish to help better those not-on-track to recovery. What constitutes positive outcomes for these patients is not fully understood.

**Aims:**

To contribute knowledge into what constitutes meaningful outcome concepts in the experiences of patients with long and complex mental health suffering and treatment, and the clinicians who work to help them.

**Methods:**

A qualitative in-depth study of 50 participants’ experiences. Data are collected through focus groups and individual interviews, and analyzed using a team based structured thematic analytic approach.

**Results:**

We found an overarching theme of outcome as an ongoing process of recovery, with the four constituent themes: (1) strengthening approach patterns for new coping; (2) embodying change reflected by others; (3) using new understandings developed in dialogue; and (4) integrating collaborative acceptance.

**Conclusions:**

We discuss our findings in light of existing empirical studies and different recovery concepts, and suggest that if outcomes monitoring is to become an integral part of routine practice, it might be beneficial to integrate an understanding of outcomes as ongoing processes of recovery within mental health suffering into these systems.

## Background

The surge of interest in routine outcome monitoring (ROM) and clinical feedback systems (CFS) in the field of mental health [1–8] re-raises some important, but also difficult questions: What is a good and relevant outcome of mental health treatment? For whom, and by whom, are outcomes defined?

The rationale that has legitimized efforts to develop and implement ROM and CFS systems in naturalistic settings has been built on the evidence that the effect of ROM and CFS is particularly pronounced for people who are *not*-*on*-*track* to improvement or recovery, and that rates of deterioration are heavily reduced [7]. This is a notion that is widely endorsed [9, 10], and that is mirrored in that Lambert [6] puts prevention of failure and dropout at the heart of his argument, rather than, for example, the enhancement of treatment effects for people who are already benefitting from treatment.

The patient who comes to treatment with a discrete area of suffering, and who is helped by the collaboration with an adequate treatment provider, and then ends treatment, is not the patient that has motivated the development of ROM/CFS. Rather, a patient who has tried therapy once, twice or maybe more times, who has maybe experienced bed unit treatment at occasions, and who has become disappointed and demoralized by not being helped, he or she is more likely motivating innovations to help prevent treatment failure. However, are relevant outcomes the same for this person, as for the person with the more short-term linear recovery process?

What is a good outcome in mental health treatment settings, considered from the patient perspective? Connolly and Strupp [11] analyzed the responses of 80 patients in the Vanderbilt Psychotherapy Research Project [12] and found 90 initial patient reported outcomes. From these, they abstracted the clusters of (a) improved symptoms, (b) improved self-understanding, (c) improved self-confidence, and (d) greater self-definition. Klein and Elliott [13] analyzed 107 client change interviews, collected from 40 patients undergoing process experiential therapies. Data collection points were during therapy and at 6- and 18-month follow up. Results show that under the two overarching domains *changes within the self* and *changes in situation*, the authors report the patient reported outcome clusters of (a) affective change, (b) self-improvement, (c) experiential processing, (d) life functioning, and (e) interpersonal relationships. Each cluster consists of parts that notably are fitting Connolly and Strupp’s [11] themes, except that Klein and Elliott’s [13] results focus more on better experiential/emotional processing and higher interpersonal trust and acceptance. Outcomes that relate to stronger agency and greater autonomy, such as Connolly and Strupps *improved self*-*definition* and *improved self*-*confidence,* and Klein and Elliott’s *life functioning,* are present in both studies, but not sorted under that theme in themselves. Moreover, Binder et al. [14] collected experiences of 10 former psychotherapy patients through in-depth interviews, and studied what constitutes good outcomes. They report four clusters or themes from their analyses, (a) new ways of relating to others, (b) reduced symptomatic distress, (c) better self-understanding/insight, and (d) acceptance and value of self.

Between the three of these studies, it is apparent that, from patients’ experiences, symptom relief is but one of four or five overarching relevant domains in considering what a meaningful outcome concept is. Nonetheless, the point that Connolly and Strupp makes [11], and that is iterated by Binder et al. [14], that psychotherapy research has strongly emphasized and continue to overemphasize discrete symptomatic relief in common outcome measures, seems as important as ever.

Particularly so now, we argue, as continuous outcomes monitoring is becoming a greater part of routine clinical practice. Studies of the patients’ perspectives on outcome cited above are tied to a psychotherapy research paradigm, whereas a substantial group of patients in ordinary clinical settings will have psychotherapy as only one of several contexts in their collaboration with the treatment provider. ROM/CFS are substantially motivated by helping better the patient group most vulnerable to drop-out and deterioration. This is a group with complex suffering who also often take part in bed unit treatment, community based approaches and integrative treatment, and for whom outcome might not simply be conceptualized by getting rid of symptoms.

Solstad et al. [15] meta-synthesized existing qualitative literature on the patient perspective on outcomes monitoring, and report that results of the included 16 studies converge around, as one of four meta-themes, that outcome monitoring needs to capture complexity. Outcome measures might generally be overemphasizing symptomatic distress, and underemphasizing situational, functional and contextual domains of outcomes. This possible bias points toward a need for developing more knowledge regarding meaningful outcome concepts for patients with more complex and long-standing suffering. A similar point is made by Friedlander et al. [16] in their discussion of treatment processes with patients with severe mental disorders. If ROM/CFS is going to be implemented broadly in naturalistic settings, people with these kinds of suffering need to be part of the picture.

On this background, we found the need to study the research questions: What do people, who have experience as patients in public mental health clinics, who have had multiple efforts at individual therapy processes, and who have had multiple experiences from hospitalized treatment, experience as meaningful positive outcomes? What do clinicians, individual therapists and specialized ward nurses, experience as meaningful good outcomes for these groups of people?

## Methods

### Methodological approach

Epistemological correspondence between what is to be studied and the method one studies it by is vital to the quality, relevance and validity of any scientific claim [17]. In this project we aim to explore experiences to discover phenomena relevant to our question about what constitutes good outcomes in public mental health settings. As such, the study is phenomenological [18] in its open exploratory and experiential focus. However, our access to explore these experiences is through language in different interview formats. What is generally provided in in-depth interviews are personal narratives and symbolized experiences. This kind of knowledge is hermeneutic [19]. This study thus has both hermeneutic and phenomenological aspects, and must therefore sail under hermeneutic-phenomenological epistemological flags [20, 21]. In the practical process of carrying out a study, the phenomenological elements are most pronounced in preparatory phases, as attitudes of openness, and in the relational meeting of interviews, whereas the hermeneutic elements are most pronounced during transcription and analytic phases [22].

### Setting

The study was organized from Helse Førde Health Trust, a public hospital trust in the western region of Norway. Førde Health Trust provides specialized somatic and mental health services to the general population in the region, comprises multiple clinical sites and has about 3000 employees. Data was primarily collected from three sites at the Department of Mental Health at the District General Hospital of Førde, Norway. Data from a reference focus group was collected at the Division of Mental Health and Addictions, Oslo University Hospital, Oslo, Norway.

### Participants

To study our research questions, we chose to collect data from a variety of perspectives, and search for core themes across these perspectives. We chose to include professionals, in addition to the patients, to have multiple perspectives and experiences in the analysis of this complex field. The first-person experiences of patients are the primary data, and the clinicians’ experiences are secondary in the analyses. 50 participants contributed to this study. They were 18 patients (six with mental health problems in one focus group, seven with mental health and addiction problems in one focus group, and five with mental health problems in individual in-depth interviews), 12 specialized psychiatric nurses, and 20 individual therapists (six psychiatrists, eight clinical psychologists, six individual therapists with other education).

We recruited patients to the study by sending an invitational letter to individual therapists in a public mental health clinic consisting of two outpatient clinics and four hospital bed units. The letter stated the purpose and design of the study explicitly. Along with this letter, therapists were asked to inform and invite patients they met to the study. As we aimed to study perspectives of a heterogeneous sample of patients, we established wide inclusion criteria: Any patient, who was presently in ongoing treatment for a mental health and or addiction problem at the District General Hospital of Førde could be included, if they based on full information experienced that they wanted to be interviewed, either in focus group the setting or the individual interview. One exclusion criteria was used to ensure participant safety: Patients who were actively psychotic at the time of the study could not be invited. Having a diagnosis of a psychiatric disorder with psychotic symptoms was not an exclusion criterion in itself, if the patient was in a stable non-psychotic phase. As the study aims for a heterogeneous trans diagnostic sample to explore and analyze common themes, a detailed collection of diagnostic information was not registered. As a group, patient participants in this study had experiences with the more severe end of mental health suffering, with experiences of both outpatient and hospitalized treatment modalities.

We recruited professionals to focus groups in the study by sending an invitational email to a convenience sample of therapists and specialized ward psychiatric nurses in the same public mental health clinic as we recruited patients from. For a reference perspective, we recruited a convenience sample of professionals for one focus group from a clinic in another part of the country. Table 1 gives an overview of participant characteristics.

**Table 1:** Sample characteristics

	Variable	Frequency
Clinicans		
Sex	Male	10
	Female	22
Age (years)	20–40	4
	41–60	28
Years practicing	>5	2
	6–15	17
	16–30	13
Type of clinician	Clinical specialist psychologist	3
	Resident psychologist	4
	Psychiatrist	5
	Resident doctor	1
	Specialized psychiatric nurse	15
	Specialized psychiatric social worker	4
Patients		
Sex	Male	7
	Female	11
Age (years)	20–40	8
	41–60	9
	61–80	1
Years experience with being a patient	6–10	7
	11–20	6
	21–30	4
	31–40	1

### Researchers

CM is a clinical psychologist with nine years of experience. He holds a position as chief advisor at a public mental health clinic where he also is head of the research group for mental health research and leads multiple research projects, and he holds adjunct positions as associate professor at the Department of Clinical Psychology, University of Bergen, Norway and the Department of Health Science, Sogn og Fjordane University College. JS and JCN are clinical psychologists at the District General Hospital of Førde, Norway, and Oslo University Hospital, Oslo, Norway, respectively. ÅS is an expert-by-experience co-researcher working for the research group led by CM, contributing to many of the group’s projects. MV is a clinical psychologist and associate professor at the Department of Clinical Psychology, University of Bergen, Norway.

Although the participating researchers’ specific focuses vary, a shared interest in humanistic, integrative and relationally oriented approaches to mental health, and real service user participation in research and clinical settings is a common ground.

### Data collection method

The main strategy for data collection in this study is the focus groups. Focus groups are a well-known strategy for collecting qualitative data [23–25]. Focus group interviews are considered beneficial in exploratory studies where researchers aim to allow the participants to build on and develop each other’s understanding [23].

Of particular importance to us in this study was to allow invited patients to voice the experiences in a way that felt safe enough for them. Since meeting with and contributing to a group interview setting is anxiety provoking for many, we also chose to offer individual interviews to patients. Five participant patients chose this option over focus groups. Individual interviews are the most common way of in-depth qualitative data collection [22, 26, 27], and provide excellent opportunities for in-depth exploration of lived experiences.

For the different interview settings, (a) focus groups with professionals, (b) focus groups with patients, and (c) individual interviews with patients, we developed interview schedules for semi-structured interviews. We aimed to balance the need for structure, that is, to make sure the interviews get at experiences that are useful in answering the research questions and that are similar enough across different interviews to allow for analyses across accounts, with the need for flexible openness, to follow the unforeseeable but interesting experiences of participants.

CM moderated three of the focus groups, the ÅS and MV moderated two focus groups and five individual interviews, JS and JCN moderated one focus group, and one focus group was moderated by a psychiatrist not authoring this paper. In sum the study builds on seven focus group interviews lasting from 1:45 to 2:10 h, including 32 professionals and 13 patients, and on five individual interviews with five patients, lasting from 37 to 72 min. We transcribed all focus group interviews and individual interviews verbatim for analyses. The full data material brought into the data analysis phase of this study thus consisted of 272 pages of single spacing transcribed text.

### Data analysis

We analyzed the data through a team-based structured approach to thematic analysis [22, 28], moving through four analytic steps aiming to discover and formulate consensual themes or meaning patterns across the data material. We build on the five concepts of consensual data analysis as presented in Hill et al. [29], namely (a) open-ended semi-structured interview schedules, (b) several analytic judges, (c) consensus about striving for thematic meaning, (d) a critical auditor, and (e) domains and cross-analyses performed. In this process, MV performed the role of the critical auditor [22, 29].

Concretely, the four steps of data analysis were: (1) CM, ÅS, JS and JCN individually read all the transcribed material closely to get a basic sense of the meanings, and made associative notes of possible themes or meaning patterns in the material, (2) CM, ÅS, JS and JCN met at a 2-day analytic seminar and worked toward consensually moving associations from the first individual readings to preliminary thematic structures, (3) after the analytic seminar, CM, ÅS, JS and JCN divided the full data material in equal parts and re-read in-depth for three weeks with the whole preliminary thematic structure in mind, to check for correspondence, (4) the resulting thematic structure with illustrative quotes was presented to MV who critically audited it. After MV’s audit, changes were made to all the four themes.

Two separate research questions were explored in this research project. The other research question, aimed concretely at the experiences of-, and needs toward, CFSs, has been reported in a separate paper [30].

### Ethical considerations

In its scope this study is positively formulated, meaning that it addresses what positive outcome is. However, both professionals and patients who were invited to contribute to the study were asked to talk from their own concrete experiences with suffering and recovery. This might lead participants toward vulnerable and sensitive personal experiences. We were highly mindful of this in planning and carrying out the study, exemplified for example through the option of the individual interviews and offering debriefing after focus group. The project was submitted to the Regional Committees for Medical and Health Research Ethics (REC) for consideration. It was there exempted from a full formal consideration (REC 2015/620-4).

## Results

In this paper we report themes that were common in the experiences between both patient participants and professional participants. In our reporting we aim to give priority to the voice of the patients, but also include an illustrative professional quote under each theme to show this line of convergence. Across the participants’ narratives and experiences, outcomes of mental health interventions were discussed and formulated as ongoing processes, rather than something that could be finished or ended. Similarly, when the participants discussed the meaning of *having become better, being well, or having an easier situation,* they stressed that these were *ever ongoing processes.* Thus, an important overall finding is that participants discussed outcomes, they described recovery more as a verb than a noun. It is something people keep on doing rather than something they have or have done. The following quote can serve illustrate how these discussions generally were verbalized:

I wouldn’t say that I’m well, from all my mental problems. But I am better, and I am doing very fine now and… yes. Being completely well, I don’t know… I wouldn’t use that word about it. I don’t think so. No.

Positive outcome experiences were talked about as living recovery processes. This overarching theme was reflected in four constituent sub-themes that represented variations and more specific processes. To represent the data closely we have chosen process formulations in naming the constituent sub-themes. The sub-themes that we found across accounts in our material were general, meaning that experiences underlying them were present in all or all-but-one interview or focus group. The themes were: (1) strengthening approach patterns for new coping; (2) embodying change reflected by others; (3) using new understandings developed in dialogue; and (4) integrating a collaborative acceptance. We detail the resulting sub-themes in the following.

## Strengthening approach patterns for new coping

In the interviews, participants emphasized how change and improvement is about finding and consolidating ways of handling the distress one may face in one’s day to day life. We have called this theme “strengthening approach patterns for new coping” to communicate how this seemed to be an active process where people themselves strive to handle their problems and challenges. That is, both participants with first-person experiences of mental distress and participant therapists highlighted the value of developing new as well as consolidating existing coping strategies. Many of them described this as a demanding process where it is the small things that count. One of them gave for example voice to the gradual aspects of this process:

Little by little my focus changed, and I changed my point of view and I got better, right. I am more capable of handling challenges that I face, and that is what I mean by getting better.

An important piece to this process was to keep on trying. All participants discussed the importance of the person’s own determination and his or her continued efforts. This quote can illustrate:

Before I was paralyzed by needing to hit the target spot on… but then I realized that the most important thing for me was just to keep on shooting those arrows, and managing to pick myself up and shoot, and if I hit something that’s great, but that I can actually define where I want my arrow to hit. Yes. So. Those kinds of insights… I feel they give me so much coping… and… simply health.

Similarly, one of the therapists that we interviewed described in the focus group discussion how change and improvement consists of an ongoing process of being in contact with important aspects of his or her life. The following quote serves to illustrate this process:

To relate to both what is good and what is hard, and that there is more a connection between what’s inside and what’s outside, that is, what one feels the need for. What one wants to do, what one wants in life. And that one is free to act on that.

## Embodying change reflected by others

In the experiences of our participants, recovery outcomes and processes seemed highly complex phenomena. Despite relying heavily on one’s own efforts and what the person who suffers actually does to handle the distress he or she faces in his or her own life, our participants simultaneously underscored how these actions unfold within a relational context. In our analyses, we were struck by how important others were, in noticing improvement and positive change, in a way that the suffering person could embody. We labeled the second sub-theme “embodying change reflected by others”. Participants emphasized the significance of acknowledging and having faith in feedback from their surroundings, as illustrated by these quotes:

So, it is those around you who see the positive change first… before you experience it yourself. So maybe it is largely about starting to trust the people around you… that… what they observe is right.

Change comes slowly but definitely, but I only see it after the fact, or maybe somebody else sees it before me and only then do I realize that this is something we’ve been working on over time.

Participants also discussed the value of input and feedback from significant others in relation to processes they had experienced in therapy. Many had experienced this as a particularly helpful element in treatment, as illustrated in the following two brief quotes emphasizing the meaning of being reminded of their own recovery processes:

Yes, it is important the therapist keeps reminding you about what is going well. And when you are all the way down, to remind you… what is happening right then and… to make you aware.

Well, what I mean a bit is that it is in the good periods, I find it very nice when she reminds me… Because then I tend to go back home and think about what we talked about.

The therapists we interviewed also emphasized the potential value of having an outsider perspective on processes of improvement and change. As these processes often are made of small, unnoticeable steps, we may need another person’s perspective to become aware of our own progression:

What is very potent in change processes I feel, is just what we have been discussing now, to be able to show to that “then we were there, now we are here—so what is this you have been achieving in the meantime, what change have we achieved together in the meantime”. Kind of having that clarified and emphasized.

An important point, however, is that this is a mutual process where the person also needs to be open to feedback and input. It is not a one-directional process in which the person who is suffering is directed by his or her significant others, but a shared process in which processes of approach and trust develop, and open up for other perspectives. One participant explored how this process of detecting change in self through the eyes of others could also be a movement towards creating positive change within herself. She described in the interview how a baby boy in her family had a very catching laughter that had an impact on her inner life and view of herself:

He has recently learned how to laugh, and when he laughs it’s impossible not to be affected by it and feel kind of like “you’re adorable” and feel some kind of good feeling inside of you and… That feeling gives me—or I have thought for a long time that I will never have such feelings again, and then I do feel it, and think that “wow, I didn’t think this was possible. What else is possible?”

## Using new understandings developed in dialogue

An important element in recovery processes and outcomes for our participants was the development of new understandings. Understandings, in the context of recovery, could make suffering easier to live with and accept. Understanding seemed to re-establish a point of view from which one could choose whether to act on difficult situations and experiences, rather than being imprisoned by them. In our interviews, both people with first-person experiences of mental distress and participants working as therapists highlighted the interpersonal nature of this kind of self-knowledge. We therefore termed our third theme “using new understandings developed in dialogue”. Here is one quote illustrating this sub-theme:

I very much needed somebody who could help me understand what was going on, and someone I could hold on to. Simply that.

Participants related these experiences to knowledge co-created in treatment, which were also collaborative and dialogical in nature. Two quotes serve as illustration:

When I was describing feelings, for example, right, and frustration and chaos, then he could sketch it up and send it back to me, what is going on and why I react as I react, and where do I need to choose another way instead of going into that tunnel and back home to bed… eh… Just being made aware like that.

I have a lot of thoughts that I shouldn’t have. That I have been having for 25 years, to be completely honest. I can’t get them away. And then I think it helps some to use… like I was saying before, kind of using her (the therapist’s) thinking a little bit, sort of. I don’t know how to explain it, but… It is about managing to turn it around. Turn around the thinking.

Relational context aside, it seems important in our material that the person struggling with his or her mental distress in the end needed to be the one to put this knowledge to use. As exemplified by the following quote, the agent in this process is he or she who battles the mental illness:

That it is not just a lot of words, but… with a little help at systematization and to understand, eh… and then I can analyze it, and… do it myself too. And I think that is an important part [leading to positive change]… it feels like I have gotten keys now, and can unlock myself.

Mirroring the experiences of people with patient experiences, therapist participants also highlighted the process of creating order and new understanding through dialogue as part of recovery, as illustrated by this quote.

Relate both to the good and the bad, and creating a connection between the inside and the outside world. What do you need, what do you wish for, what do you want from life? And having the possibility to act on that, and not just the other things.. I find the word integration really important.. an integrated person, a developed… a developed sense of health is about being able to let your guard down when it fits, and hold your guard up when that fits, I think… Sharing feelings when that fits, and sometimes holding it in.

## Integrating a collaborative acceptance

We named the fourth theme “integrating a collaborative acceptance”, because all participants highlighted recovery outcomes and processes as stemming from human encounters built on fundamental recognition and acceptance. Recognition provides a way in which collaboration helps bring the person forth. In our sample, participants described these processes independent of specific patient needs or therapist strategies that make up this collaboration on a formal level. The following quote illustrates some of the qualities in this theme:

It is kind of like you have a therapist who manages to be present with just small things the day when nothing really works for you. And then gee, you might end up with a smile on your face leaving the therapist office, because it’s just like… it is okay to relax in sessions. That you are allowed to just be who you are, from time to time.

While it seemed the process of recognition is what makes the person come forth in recovery processes, rather than surrendering to a patient role or diagnostic category, warm acceptance seemed to be part of the same process. This illustrative quote is from a therapist focus group:

It is immensely important that the health care provider says, early in the process, that here you can talk about anything, and that here, nothing will be forgotten, or something like that. Here, we don’t pass judgements or look down our noses or anything.

Many of the participants described developing recognition and acceptance as an inherently collaborative process, going on both in actual meetings and between meeting points in the treatment process. Two quotes can illustrate this:

For me it is about hearing the therapist’s voice a little. I sort of hear it afterwards. And it helps me in the things that I shall perform and do. Then I kind of use…

If it hadn’t worked [treatment] I wouldn’t be sitting here today. I simply wouldn’t have been here. Hadn’t it helped, hadn’t here been this one person who would carry hope for me until I was able to carry it for myself… And it is not even that long ago… It is not that many years ago.

In the experiences of our participants, the processes of recognition and acceptance seemed to build on a fundamental quality of care, being cared for and daring to care for oneself. Both being cared for and taking care of oneself are described in ways that suggest a collaboration, a getting-to-know-me that requires a back-and-forth between the collaborating partners over a period of time. Furthermore, this seemed to strengthen the feeling of not being given up on—instead being recognized, accepted, and mutually cared for. From what many of the participants told us, it occurs that this process was about establishing a groundwork for good outcomes. One therapist quote illustrates a general summary of these discussions:

… to have been listened to… to have been followed up on, to have been cheered for and… well, yes, been taken seriously.

## Discussion

This study comprises dual perspectives on experiences of outcomes from people with complex patient experiences and clinicians who work to provide care and treatment for them in the ordinary clinic. One important quality of the results seems to be that every constituent theme understands outcome as ongoing processes for these people. Moreover, an important relational context or a collaborative process of achieving outcomes is evident in all the reported themes, mirroring the results reported by Klein and Elliott [13] focusing on life functioning and improved interpersonal relationships. Indeed, self-acceptance, self-understanding, improved interpersonal safety and functioning converge as important descriptors of what constitutes positive outcome both in the existing qualitative studies reported [11, 13, 14] and in our study. However, a common denominator between our relationally laden themes is the importance of patient agency, that is, doing things rather than being done things to. In this sense, our findings reflect qualities inherent in, in particular, the categories of improved self-understanding, improved self-confidence, and greater self-definition, in the results reported by Connolly and Strupp [11].

Our findings differ from the study by Binder et al. [14] in that symptoms were very rarely autonomously pointed to by participants when discussing outcome experiences. When prompted in the interviews, the typical response was briefly agreeing to that it was important, then moving on to describing other experiences with more engagement. We think we should be careful in interpreting this, and in any way we should not therefore think that for our participants achieving improved symptomatic states was unimportant. Rather, it seems that symptoms are understood as part of more important phenomena, such as how one functions with family, children, or at work. Function, that is, how one is *doing*, seemed more important than how one was *feeling,* in our material. In this sense, our findings underscore the value of what we may call a social agency [31, 32] for recovery processes and outcomes.

In the recovery literature, a conceptual difference if often explained by contrasting *recovery within* to *recovery from* a mental health problem [33, 34]. *Recovery from* refers to understanding mental health suffering within a medical model, as discrete illnesses that display symptoms, and that have their end-point when symptoms are removed. *Recovery within* refers to mental health suffering and illness as parts of experiences that constitute challenges, burdens, obstacles for the person trying to live well, but that health comes from finding meaningful ways of living with, rather than getting rid of, these experiences. In the latter understanding of recovery, end points are less meaningful and symptoms are subordinate to live and living functions [35].

In concordance with the concept of *recovery within*, an abundant empirical literature builds on the construct *personal recovery*, defined as “a deeply personal, unique process of changing one’s attitudes, values, feelings, goals, skills and/or role. It is a way of living a satisfying, hopeful and contributing life even with the limitations caused by illness” [36]. In a systematic review of personal recovery, Leamy et al. [37] synthesized results from 97 papers and proposed the acronym CHIME, comprising the five general recovery processes of (a) connectedness, (b) hope and optimism, (c) identity, (d) meaning in life and (e) empowerment. They underscored that recovery is an active, individual and unique process. Clearly, our results fit the *recovery within* and *personal recovery* model better than the *recovery from*. One apparent understanding of this is that we deliberately invited people with complex treatment histories and suffering and the people who try to help them, to this study, to understand what positive outcomes are, giving voice to complex personal change narratives.

However, understanding how to integrate this active and unique conceptual understanding of recovery into routine standardized measures of outcome of mental health services remains a priority. A systematic review [38] found 13 personal recovery measures and evaluated nine for psychometric properties and 12 for conceptual fit with the CHIME [37] processes. They concluded that no recovery measures could yet be unequivocally be recommended; in particular due to lack of information on feasibility, time to complete, responsiveness, construct validity and criterion validity. Developing routine outcome measures (ROM) that integrate researchers’ and providers’ needs for standardization with patients’ needs for personalization calls for continuous conceptual and empirical efforts. Elsewhere, we have reported empirical and conceptual work to argue that using new technology to conceptualize ROM as dynamic systems of patient self-report for immediate clinical feedback rather than static measures might be one way of moving forward in these efforts [30].

### Implications

A key finding in our study is that recovery outcomes and processes are experienced as diverse and complex phenomena—both depending on the person’s own efforts to promote positive change in his or her life and at the same time being embedded within a relational context. One implication that comes out of this is the importance of developing partnerships that are based on, and closely linked to, the individual’s own coping strategies, in working to help these people. A second implication is that if routine outcome monitoring is to be implemented broadly, ways of detecting changes beyond symptom reduction, such as relational functioning, self-understanding, and agency need to be integrated into the measures.

### Limitations and strengths

A major strength in the present study is its ability to approach outcomes and processes within a design that builds on a variety of experiences. We have aimed to understand these experiences from both the perspective of clinicians with professional background and from the first-person perspective of those having experienced processes of recovery in the context of battling a mental health problem. Convergence between different perspectives might be considered an indication of conceptual validity of findings. We consider this multiplicity of experiences and point of views as holding potential of allowing the research questions to be approached in new and different ways [39, 40].

A possible limitation to the study is a potential selection bias, arising from the fact that clinicians judged which patients to invite to the study. This could have led the clinicians to invite patients with a particular recovery profile and consequently narrowing the sample. However, efforts were taken to balance this potential bias: The invitation to the study clearly stated that the project aimed to explore a broad range of experiences. We can think of now overt incentives for clinicians to bias their selection to any particular kind of patient.

### Researcher reflexivity

A limitation of the present study is that all participants are drawn from the same region that the researchers work. The researchers’ clinical profile might have been known to participants, or the participants might have shared cultural affiliations that made the sample more homogenous than beneficial to an explorative study. One effort to counter this potential bias is our inclusion of a reference focus group from another institution in a different part of the country. Moreover, although care has been taken to work reflexively [41, 42] in carrying out this study, the researchers’ professional interest will have had an impact on how interviews were performed. We have worked to balance potential biases by including a critical auditor to the analyses who had not been part of data collection and early analyses. Moreover, we have explicitly worked in group based analysis to make explicit potential blind spots of each researcher.

To increase the credibility of the results we have worked to present the study with a high degree of transparency in describing the involved researchers and study settings, detailing the concrete steps of the study, and providing a fair amount of illustrative quotes to give the reader a chance to follow analyses. With these undertakings we aimed to establish grounds for readers to evaluate the trustworthiness [43] of the results.

One solution to limitations coming from the situatedness of the study and the researchers could be if similar research questions could be addressed by different researchers in different context with a similar sample of participants.

## Conclusion

In this qualitative study of 50 persons’ perspectives on recovery processes and outcomes, we found the following four themes when analyzing their points of view on what constitutes improvement and positive change: (1) strengthening approach patterns for new coping; (2) embodying change reflected by others; (3) using new understandings developed in dialogue; and (4) integrating collaborative acceptance. If outcomes monitoring is to become an integral part of routine practice, it might be beneficial to integrate an understanding of outcomes as ongoing processes of recovery into these systems.

## Appendix

See Table 1.

## Abbreviations

ROM: routine outcome monitoring
CFS: clinical feedback systems
